# Interdisziplinäre multimodale Schmerztherapie bei postviralen Syndromen und ME/CFS: Besonderheiten, Fallstricke und Modellkonzept

**DOI:** 10.1007/s00482-023-00761-2

**Published:** 2023-10-20

**Authors:** Benjamin Luchting, Uta Behrends, Bianca Eigner, Silvia Stojanov, Cordula Warlitz, Matthias Haegele, Eva Neuwirth, Lorenz Mihatsch, Hans Peter Richter

**Affiliations:** 1 Interdisziplinäre Schmerztagesklinik der München Klinik Schwabing, Klinik für Anästhesiologie, Kinderanästhesiologie und Operative Intensivmedizin, München Klinik Schwabing, München, Deutschland; 2 MRI Chronische Fatigue Centrum für junge Menschen. Zentrum für Kinder- und Jugendmedizin, Technische Universität München und München Klinik, München, Deutschland; 3 Klinik und Poliklinik für Anästhesiologie und Intensivmedizin, Technische Universität München, München, Deutschland

**Keywords:** Schmerzen, Post-COVID/Long-COVID, Myalgische Enzephalomyelitis/Chronisches Fatigue-Syndrom, Belastungsintoleranz, Behandlungskonzept, Pain, Post-COVID/Long-COVID, Myalgic encephalomyelitis/chronic fatigue syndrome, Stress intolerance, Therapy concept

## Abstract

**Hintergrund:**

Multimodale Schmerztherapien erfolgen üblicherweise im Rahmen von mehrwöchigen Gruppentherapien und basieren auf einem generell aktivierenden Ansatz. Durch die Besonderheit einer Belastungsintoleranz mit postexertioneller Malaise (PEM) bei Patient:innen mit postviralen Syndromen muss in diesen Fällen eine körperliche sowie psychische Überlastung dringend vermieden werden. Diese Aspekte können in gängigen schmerzmedizinischen Therapiekonzepten jedoch nur unzureichend berücksichtigt werden.

**Methodik:**

Zusammenfassung der aktuellen Literatur und Darstellung klinischer Besonderheiten sowie Vorstellung eines therapeutischen Modellprojekts für eine interdisziplinäre multimodale Schmerztherapie bei postviralen Syndromen mit PEM.

**Modellkonzept:**

Das vorgestellte Modellkonzept beschreibt ein der individuellen Belastbarkeit angepasstes tagesklinisches Behandlungssetting für die multimodale Schmerztherapie mit Minimierung des Risikos einer belastungsinduzierten Zustandsverschlechterung.

## Abstract (en)

**Background:**

Multimodal pain therapy usually take place in the context of group therapy lasting several weeks and is based on a generally activating approach. Due to the specificity of stress intolerance with postexertional malaise (PEM) in patients with postviral syndromes, physical as well as psychological overload must be urgently avoided in these cases; however, these aspects can only be insufficiently considered in current medical pain therapy concepts.

**Methods:**

Summary of the current literature and presentation of clinical characteristics as well as presentation of a model project for a multimodal pain therapy in postviral syndromes with PEM.

**Model concept:**

The presented model project describes a day clinic treatment setting for interdisciplinary multimodal pain therapy adapted to the individual resilience with minimization of the risk of strain-induced deterioration of the condition.

Bei der Behandlung von Patient:innen mit chronischen Schmerzen im Rahmen postviraler Syndrome oder einer myalgischen Enzephalomyelitis/eines chronischen Fatigue-Syndroms (ME/CFS) können Therapiemaßnahmen ohne Berücksichtigung einer möglichen Belastungsintoleranz mit postexertioneller Malaise (PEM) zu schwerwiegenden chronischen Verläufen und einer Verschlechterung des Allgemeinzustands bis hin zur Bettlägerigkeit führen. Unter anderem aufgrund dieser Besonderheit müssen bei der Durchführung interdisziplinärer multimodaler Schmerztherapien (IMST) besondere Aspekte beachtet werden. Maßnahmen zur körperlichen, kognitiven oder psychischen Aktivierung sollten im Falle von PEM abhängig vom individuellen Ausgangsbefund vermieden oder nur im Rahmen eines konsequenten, vorausschauenden Energiemanagements (Pacings) erfolgen. Trotz der hohen Anzahl an Betroffenen mit chronischen Schmerzen im Rahmen von schweren postviralen Syndromen und ME/CFS gibt es bislang kaum schmerzmedizinische Einrichtungen mit einem passenden Therapieangebot.

Im Rahmen der COVID-19-Pandemie entwickelten Schätzungen zufolge bis zu 15 % aller Personen, die mit SARS-CoV‑2 infiziert waren, lang anhaltende oder wiederkehrende Symptome, die unter dem Begriff „Long-COVID“ oder „Post-COVID-Syndrom“ (PCS) zusammengefasst wurden [12]. Die Prävalenz von Long-COVID hat mit dem Auftreten der Omicron-Varianten deutlich abgenommen. Sie liegt derzeit gemäß einem Survey in UK bei knapp 3 % der Gesamtbevölkerung und niedriger bei Kindern und Jugendlichen [25]. Weiteren Schätzungen zufolge sind allein in den USA ca. 1 Mio. Menschen aufgrund eines PCS erwerbsunfähig und die Gesamtkostenschätzung für die USA (Stand 10/2021) liegt bei 2,6 Billionen US-Dollar [5]. In Deutschland liegt die durchschnittliche Arbeitsunfähigkeit bei Long-COVID mit 105 Tagen deutlich über dem allgemeinen Durchschnitt von 14,6 Tagen/Jahr [11].

Einige von PCS Betroffene erfüllen die Diagnosekriterien für die myalgische Enzephalomyelitis/das chronische Fatigue-Syndrom (ME/CFS), welche bzw. welches als die schwerste Verlaufsform postviraler Syndrome gilt [32]. Die geschätzte Inzidenz von ME/CFS war im Jahr 2020 nach dokumentierter SARS-CoV-2-Infektion gemäß deutschen Krankenkassendaten höher als ohne dokumentierte Infektion (0,6 % versus 0,2 %; [30]). Laut einer Stellungnahme der Kassenärztlichen Bundesvereinigung lag die Gesamtprävalenz von ME/CFS in Deutschland im Jahr 2018/2019 bei 350.000–400.000 und im Jahr 2021 bei fast 500.000 Fällen [16]. In spezialisierten Ambulanzen wurden 13–49 % PCS-Betroffene mit ME/CFS diagnostiziert [2, 8, 17], am MRI Chronische Fatigue Centrum für junge Menschen (MCFC) der Technischen Universität München (TUM) auch erste Kinder und Jugendliche [26].

Neben zahlreichen unspezifischen Symptomen wie krankhafter Erschöpfung (Fatigue), kognitiven Defiziten („brain fog“), Schlafstörungen oder Kreislaufproblemen stellen auch anhaltende Kopf‑, Muskel- und/oder neuropathisch anmutende Schmerzsyndrome sehr häufige Beschwerden dar [19]. Leitsymptom von ME/CFS ist eine Belastungsintoleranz mit lang anhaltender, oft versetzt eintretender Symptomverschlechterung nach bereits geringfügigen Alltagsbelastungen, die vor der Erkrankung problemlos toleriert wurden (sogenannte postexertionelle Malaise, kurz PEM; [15]).

Trotz aktueller, kontroverser Diskussionen über die Zuordnung der multisystemischen Erkrankung zum eher somatischen oder psychosomatischen Formenkreis [14] besteht ein fächerübergreifender Konsens, dass deren Behandlung einen interdisziplinären, multimodalen Therapieansatz erfordert [13, 31]. Während es deutschlandweit inzwischen bereits über 100 PCS-Ambulanzen gibt, liegt dort der Fokus meist auf der umfangreichen Diagnostik. Therapeutische Einrichtungen für eine adäquate Versorgung und nachhaltige medizinische Betreuung sind weiterhin rar [34].

In der Literatur finden sich bereits einige Beobachtungsstudien und Übersichtsarbeiten zu unterschiedlichen Rehabilitationsansätzen, bislang jedoch keine Leitlinie hinsichtlich IMST bei postviralen Syndromen. Die seit Jahren etablierten IMST-Konzepte sowohl in der Schmerz- und Rehabilitations- als auch in der psychosomatischen Medizin sollten eigentlich die ideale Grundstruktur für eine adäquate Versorgung bieten. Grundlage dieser Behandlungskonzepte ist allerdings ein generell aktivierender Ansatz, welcher bei postviralen Erkrankungen häufig nicht zielführend oder sogar kontraindiziert sein kann: Durch die Besonderheit beispielsweise der belastungsinduzierten Erschöpfung mit der Gefahr einer anhaltenden PEM muss eine körperliche, kognitive und psychische Überlastung dringend vermieden werden [33]. Die Belastbarkeit der Betroffenen ist häufig so stark eingeschränkt, dass bereits ein Assessment- bzw. Behandlungstag einen „Crash“ auslösen kann mit einer eventuell langfristigen Zustandsverschlechterung.

## Ziel

Die Betroffenen befinden sich aktuell in einem Dilemma: Hinsichtlich postviraler Syndrome und ME/CFS gibt es bis dato keine kurative Therapie, für eine adäquate Schmerzbehandlung im konventionellen IMST-Setting sind sie häufig nicht belastbar genug. Unser Anliegen ist es daher, den Betroffenen einen Zugang zu der bestehenden multiprofessionellen Infrastruktur in einer bedürfnisorientierten Form zu ermöglichen [18]. Angesichts der zahlreichen Besonderheiten bei der Behandlung von Patient:innen mit postviralen Syndromen und der Gefahr einer PEM bei zu hohen körperlichen, kognitiven oder emotionalen Anforderungen soll dieser Artikel eine entsprechende Übersicht verschaffen und potenzielle Fallstricke aufzeigen. Darüber hinaus soll die exemplarische Vorstellung eines Modellkonzepts schmerzmedizinische Einrichtungen dazu inspirieren, durch die Implementierung von IMST-Angeboten die bislang unzureichende Versorgungssituation in Deutschland zu verbessern.

Alle Empfehlungen wurden bewusst allgemein gehalten aufgrund der Tatsache, dass die verschiedenen schmerzmedizinischen Einrichtungen über breit gefächerte Therapie- und Therapeutenspektren mit unterschiedlichen Schwerpunkten verfügen und es bislang keine Daten zur Überlegenheit einzelner Therapiemodule gibt.

## Besonderheiten und Fallstricke

### Besonderheit: Belastungsintoleranz mit postexertioneller Malaise (PEM)

Eines der eindrücklichsten und gravierendsten Symptome bei schweren postviralen Syndromen oder dem Vollbild von ME/CFS stellt die belastungsabhängige Symptomverschlechterung (PEM) dar: Diese äußert sich typischerweise durch eine lang anhaltende Verschlechterung der Gesamtsymptomatik nach körperlicher, mentaler oder emotionaler Anstrengung und hält im Falle von ME/CFS mindestens bis zum Folgetag an. Ausdauer- und Muskelaufbautraining kann eine PEM auslösen [15].

Aufgrund dieser Charakteristik ergibt sich die Notwendigkeit eines achtsamen Umgangs mit den individuellen Energieressourcen sowie der strikten Vermeidung von Überlastungen, um Häufigkeit und Intensität von sogenannten Crashs zu reduzieren oder zu vermeiden. Hierfür bedarf es einer entsprechenden Strategie des Aktivitäts- und Energiemanagements, welche im internationalen Kontext als „Pacing“ bezeichnet wird. Aufgrund der großen intra- und interindividuellen Variabilität der Aktivitätsniveaus, die eine PEM auslösen können, ist es entscheidend im Rahmen des Pacings herauszufinden, welche Aktivitäten aktuell toleriert werden können, ohne dass die Symptome verschlimmert werden. Dies kann auch deswegen eine Herausforderung darstellen, weil sich die PEM sehr verzögert mit bis zu 72-stündiger Latenz einstellen kann. Zusätzlich kann die individuelle Belastungsgrenze sowohl im Krankheitsverlauf als auch tagesaktuell variieren. Aus diesem Grund ist eine ständige Anpassung der Pacing-Maßnahmen erforderlich und ein starres Rahmenprogramm nicht zielführend. Hilfreich ist beispielsweise eine fortlaufende Dokumentation (Tagebuch), um einen Zusammenhang von Alltagsaktivitäten und PEM festzustellen sowie durch vorsichtige Steigerung die individuelle Belastungsgrenze zu bestimmen. Neben gängigen Entspannungstechniken wie der progressiven Muskelrelaxation nach Jacobson bieten sich zur Ermöglichung eines sehr niedrig intensiven Körperwahrnehmungs- und Ausdauertrainings bspw. Liegend-Yoga, Feldenkrais oder Qigong an. Auch hier muss aber auf die Gefahr einer PEM Rücksicht genommen werden [31].

Überlastungen durch die psychotherapeutische Behandlung sollten ebenfalls vermieden werden: Die bisherige Studienlage zur Anwendung der kognitiven Verhaltenstherapie bei ME/CFS erscheint kontrovers und Befunde sind aufgrund methodischer Mängel und mangelnder Differenzierung von Konzepten kaum vergleichbar [14]. Die kognitive Verhaltenstherapie wird auf Basis der aktuellen Befunde zu diesem Störungsbild nicht als kurative Strategie, sondern primär zur individuellen Symptom- und Lebensbewältigung, der Belastungsreduktion und der Verbesserung der Funktions‑, Partizipations- und Handlungsmöglichkeiten im Kontext der chronischen Erkrankung empfohlen [10]. Durch supportive, entlastende Strategien wird die Erhöhung der Lebensqualität des Individuums fokussiert [37, 38]. Auch zur primären Behandlung psychischer Komorbiditäten (affektive Symptomatik wie Depressivität und Ängste, Anpassungsprobleme) bei mild oder moderat Betroffenen kann eine entlastende kognitive Verhaltenstherapie in adaptierter Form angeboten werden [20, 24]. Verfahrensübergreifend sollten dabei im Rahmen der Behandlung auch psychodynamische und systemische Gesichtspunkte Berücksichtigung finden. Nach ersten Erfahrungen mit betroffenen Patient:innen erscheint folgender Rahmen für die psychotherapeutische Arbeit sinnvoll: zeitlich angepasste (verkürzte) Behandlungen im Einzelsetting sowie räumlich angepasste Therapiesitzungen, die eine Reizüberempfindlichkeit berücksichtigen (Licht, Temperatur, Akustik, Sitz‑/Liegeposition).

### Besonderheit: orthostatische Intoleranz und posturales Tachykardiesyndrom (PoTS)

Eine häufige Komorbidität bei postviralen Syndromen und ME/CFS ist das posturale Tachykardiesyndrom (PoTS) mit einer Häufigkeit von ca. 25 % bei ME/CFS-Patient:innen [36]. Definitionsgemäß spricht man von einem PoTS bei orthostatischen Symptomen für mindestens drei Monate in Kombination mit einem anhaltenden, inadäquaten Herzfrequenzanstieg in einem angelehnten 10-Minuten-Stehtest („NASA lean test“; bei Erwachsenen um ≥ 30 bpm, bei Jugendlichen unter 19 Jahren um ≥ 40 bpm und/oder altersunabhängig auf ≥ 120 bpm) ohne orthostatische Hypotension.

Neben dem Stehtest kann zur ergänzenden Diagnostik ein Kipptischtest durchgeführt werden, der allerdings eine zusätzliche Belastung mit sich bringt und nicht überall verfügbar ist. Zur Quantifizierung verwenden wir in unserem Setting zusätzlich zum Stehtest einen konsentierten Orthostase-Fragebogen der Rheinisch-Westfälischen Technischen Hochschule (RWTH) Aachen und der Technischen Universität München (TUM).

Eine gute Übersicht über potenzielle therapeutische Maßnahmen bietet die S1-Leitlinie „Synkope“. Danach bietet sich nach Ausschöpfung der physikalischen Maßnahmen (Trinkmenge, Salztabletten, Physiotherapie/Stehtraining, Kompressionsstrümpfe) bei ausbleibender Besserung ein Off-label-Versuch mit Midodrin, Pyridostigmin oder Ivabradin an. Weiterhin können niedrig dosierte β‑Blocker hilfreich sein [6].

### Besonderheit: Fatigue, Kognition, Stressintoleranz

Ein ebenfalls häufiges Symptom bei postviralen Syndromen und essenzielles Symptom von ME/CFS stellt die anhaltende Fatigue dar. Diese wird definiert als eine „subjektiv stark einschränkende, zu den vorausgegangenen Anstrengungen unverhältnismäßige, sich durch Schlaf oder Erholung nicht ausreichend bessernde subjektive Erschöpfung auf somatischer, kognitiver und/oder psychischer Ebene“ [19]. Des Weiteren berichten bis zu 35 % der Post-COVID-Betroffenen kognitive Defizite, den sogenannten „brain fog“, welcher unter anderem Konzentrations- und Aufmerksamkeitsstörungen, ein gestörtes Kurzzeitgedächtnis sowie Wortfindungsstörungen umfasst. Weiterhin bestehen häufig Schlafstörungen sowie Stressintoleranz, insbesondere bei Reizüberflutung [40]. Der Umstand dieser stark einschränkenden Begleitsymptomatik muss im therapeutischen Setting bei allen Modulen hinsichtlich Moduldauer, Pausenzeit und Reizabschirmung unbedingt berücksichtigt werden.

### Besonderheit: Schmerzphänotyp

Schmerzen stellen ein sehr häufiges Symptom bei postviralen Syndromen dar. Eine umfangreiche Übersicht hierzu bietet der Artikel „Aktualisierte S1-Leitlinie Long/Post-COVID: Relevante Aspekte für die Schmerzmedizin“ in *Der Schmerz* 03/2023 [22]. Demnach leiden ca. 44 % der Patient:innen an anhaltenden Kopfschmerzen, gefolgt von myofaszialen, Gelenk- und Gliederschmerzen sowie neuropathisch anmutenden Schmerzen. Eine Besonderheit hierbei ist das parallele (Neu‑)Auftreten oft mehrerer dieser Schmerzsyndrome gleichzeitig und das häufige Nichtansprechen auf gängige Analgetika. In der aktuellen S1-Leitlinie wird die jeweilige Schmerztherapie abhängig von der Art der Schmerzen in Anlehnung an die jeweiligen AWMF-Leitlinien empfohlen [22]. Die Besonderheiten der Begleitfaktoren bei postviralen Syndromen bzw. ME/CFS bleiben hierbei aber unberücksichtigt.

Kopfschmerzen bei postviralen Syndromen treten entweder neu auf oder präsentieren sich durch eine Zunahme/Veränderung und/oder Häufung bereits bekannter Kopfschmerzen vermutlich ohne klinisch spezifischen Phänotyp [7]. Das klinische Bild lässt sich in einen migräne- oder spannungskopfschmerzartigen Typ unterscheiden. Häufig sind bereits die Diagnosekriterien für einen chronischen Kopfschmerz/Migräne oder neu aufgetretenen täglichen Kopfschmerz (engl. „new daily persisting headache“, kurz NDPH) erfüllt [35].

Muskel- und Gelenkschmerzen präsentieren sich oftmals multilokulär (fibromyalgieform). Neuropathisch anmutende Schmerzsyndrome manifestieren sich häufig mit Symptomen einer Dysautonomie, Polyneuropathie/Small-Fiber-Neuropathie mit Ganzkörperallodynie, multilokulären Dysästhesien und/oder Burning-mouth- oder Burning-eye-Syndrom [39].

Persistierende Halsschmerzen sind vielfach beschrieben und sprechen kaum auf gängige Analgetika an. Hierbei sollte differenzialdiagnostisch auch an eine subakute Thyreoiditis de Quervain oder aber eine potenzielle Agranulozytose gedacht werden. Anästhetikahaltige Gurgel‑/Spüllösungen können schmerzlindernd eingesetzt werden.

### Besonderheit: medikamentöse Schmerztherapie

Patient:innenberichten zufolge sind bei Kopfschmerzen zuvor wirksame Analgetika oft nicht mehr wirksam. Systematische Untersuchungen liegen kaum vor. Eine retrospektive Analyse berichtete für Post-COVID-Kopfschmerzen über eine Wirksamkeit von Indometacin (2 × 50 mg pro Tag über 5 Tage) bei zuvor ausbleibendem Ansprechen auf nichtsteroidale Antiphlogistika und Triptane [21]. Eine weitere retrospektive Untersuchung zeigte basierend auf 48 Patient:innen mit Post-COVID-Kopfschmerzen unter Behandlung mit Amitriptylin eine Reduktion der Anzahl an Kopfschmerztagen pro Monat um 9,6 Tage [9]. Bei Menschen mit postviralen Syndromen oder ME/CFS sind die Kriterien für eine chronische Migräne oder einen chronischen Spannungskopfschmerz bzw. einen neu aufgetretenen täglichen Kopfschmerz häufig erfüllt [35]. Bei der Wahl der Prophylaktika sollte Begleitsymptomen wie PEM, PoTS, Fatigue und „brain fog“ eine besondere Beachtung geschenkt werden. Während (niedrig dosierte) β‑Blocker bei PoTS gegebenenfalls einen positiven Begleiteffekt erzielen können, wirkt sich Amitriptylin eher negativ auf die Herzfrequenz aus. Bei Topiramat und Amitriptylin müssen die zentralnervösen Nebenwirkungen beachtet werden, um eine bestehende Fatigue und/oder kognitive Defizite nicht weiter zu verstärken. Möglicherweise könnte der frühzeitige Einsatz von Botulinumtoxin und/oder Antikörpern gegen „calcitonin gene-related peptide“ (CGRP) sinnvoll sein.

Auch bei der Therapie von neuropathisch anmutenden Schmerzsyndromen sollten die zentralnervösen Nebenwirkungen von Koanalgetika beachtet werden, da sich sowohl Gabapentinoide als auch Amitriptylin negativ auf kognitive Einschränkungen („brain fog“) und Fatigue auswirken können. Selektive Serotonin-Noradrenalin-Wiederaufnahmehemmer (SSNRI) wie Duloxetin sind dabei gegebenenfalls besser geeignet.

Bei begleitenden Schlafstörungen könnte die zentralnervöse Wirkung der Koanalgetika wiederum hilfreich sein, ansonsten bieten sich hier Melatonin und Antihistaminika der 1. Generation an [29]. Medikamente mit Abhängigkeitspotenzial sollen nicht regelmäßig zur Schlafinduktion eingesetzt werden.

Als eine weitere Besonderheit ist festzustellen, dass die Betroffenen häufig bereits diverse Nahrungsergänzungsmittel einnehmen, darunter vor allem Magnesium, Vitamin D, B‑Vitamine, Coenzym Q10, N‑Acetyl-Cystein, NADH, Selen und/oder Zink. Die Einnahme basiert häufig nur auf Fallberichten und erfolgt nicht selten ohne laborchemisch bestätigte Mangelsituation. RCT zur Wirksamkeit einzelner Substanzen gibt es bislang keine. Insbesondere bei dem Leitsymptom Kopfschmerz kann die Kombination von Magnesium, Vitamin B2 und Coenzym Q10 (z. B. Migravent) erwogen werden. Überdosierungen von Vitamin B6 sind bei Selbstmedikation nicht selten und sollen vermieden werden.

## Exemplarisches Modellprojekt

Um den Besonderheiten gerecht zu werden, entwickelten wir für Betroffene mit postviraler PEM und dem Leitsymptom Schmerz ein spezielles tagesklinisches, multimodales Therapieprogramm in unserer Schmerztagesklinik. Die Schmerztagesklinik der München Klinik Schwabing ist eine interdisziplinäre Einrichtung mit Behandlungsschwerpunkt auf teilstationären, nichtinvasiven, multimodalen Schmerztherapien gemäß den Empfehlungen zu Struktur- und Prozessparametern der Ad-hoc-Kommission „Interdisziplinäre Multimodale Schmerztherapie“ (IMST) der Deutschen Schmerzgesellschaft e. V. und OPS-Katalog-Code 8‑918 [1, 27]. Die Etablierung des Konzepts erfolgte in enger Kooperation mit dem MRI Chronische Fatigue Centrum für junge Menschen (MCFC) der Technischen Universität München (TUM). Die Evaluation umfasst unter anderem die Erhebung der Schmerzen, der Depressivität, der longitudinalen Symptomlast, der Alltagsfunktion und der gesundheitsbezogenen Lebensqualität. Das Pilotprojekt startete primär mit der Behandlung von Erwachsenen. Eine Erweiterung des Therapieangebots für Kinder und Jugendliche ist in Planung.

## Assessment und Therapiesetting

Im Idealfall erfolgte vor der Anmeldung für eine IMST bereits eine differenzialdiagnostische Abklärung (u. a. internistisch, kardiologisch, neurologisch, pneumologisch, psychiatrisch) sowie Diagnosestellung in einer interdisziplinären Post-COVID-Ambulanz. Aufgrund langer Wartezeiten und regional unterschiedlicher Verfügbarkeit ist dies jedoch bei vielen Anmeldungen noch nicht gegeben, weswegen große Teile der Diagnostik zu postviralen Syndromen bzw. ME/CFS im Rahmen des Schmerzassessments nachgeholt werden müssen. Da validierte diagnostische Biomarker für postvirale Syndrome und ME/CFS fehlen, erfolgt die klinische Diagnose nach gründlicher differenzialdiagnostischer Abklärung anhand international empfohlener Diagnosekriterien. Wir verwenden die vom Europäischen Netzwerk für ME/CFS (EUROMENE) und von den Centers for Disease Control and Prevention (CDC) empfohlenen Kriterien des Institute of Medicine (IOM) mit zusätzlicher Evaluation der kanadischen Konsensuskriterien (CCC; [3, 23]).

Sofern bei erster Kontaktaufnahme wegen Schmerzen Hinweise auf Fatigue, PEM und/oder ein mögliches postvirales Syndrom bestehen, sollten neben der Abfrage der Belastbarkeit und Wohnortnähe die zu versendenden Anmeldeunterlagen um spezielle Fragebögen zur Evaluation von PEM (DSQ-PEM: [4]) sowie zu den klinischen Diagnosekriterien für ME/CFS ergänzt werden [3, 23]. Die sorgfältige Evaluation von PEM und ME/CFS ist von entscheidender Bedeutung, um zu vermeiden, dass Patient:innen mit PEM einer kontraindizierten Überlastung bereits durch das Assessment ausgesetzt werden.

Der kurze Munich Berlin Symptom Questionnaire (MBSQ), mit dem beide Kriterien (CCC und IOM) gleichzeitig erhoben werden können, wurde von uns kürzlich als „preprint“ auf medRxiv publiziert [26]. Wir wenden außerdem eine eigens entwickelte Aktivitätsskala sowie einen von der RWTH Aachen und dem MCFC der TUM eigens entwickelten Orthostasefragebogen an, um den Gesundheitsstatus vorab einzuschätzen. Ergänzendes, umfangreiches Informationsmaterial zur ME/CFS wird von der Deutschen Gesellschaft für ME/CFS zur Verfügung gestellt (https://www.mecfs.de).

Bei der Durchführung des interdisziplinären Schmerzassessments (OPS 1‑910) muss dann ebenfalls die individuelle körperliche und mentale Belastbarkeit berücksichtigt werden. Unabhängig davon, ob ein eintägiges oder zweitägiges Assessment geplant wird, sollte auf alle Fälle ein reizarmer Aufenthaltsraum zur Verfügung stehen und ausreichend Pausenzeit zwischen den Visiten der verschiedenen Fachdisziplinen eingeplant werden. Bei beginnenden Anzeichen von Überlastung sollte der Assessmenttag abgebrochen werden. Gegebenenfalls kann bei deutlicher Zustandsverschlechterung auch eine stationäre Aufnahme erwogen werden, sofern die Strukturen der Einrichtung dies zulassen.

Um redundante Fragen zu vermeiden, kann es hilfreich sein, wenn beispielsweise bei der ärztlichen Diagnostik bereits weitere Fachdisziplinen anwesend sind. In unserem Modell beginnen wir mit dem ärztlichen Gespräch im Beisein der Psycho- und Physiotherapeut:innen. Nach entsprechender Pause folgt dann im Einzelsetting die psychologische Exploration und, sofern noch ausreichend Energiereserven vorhanden sind, die physiotherapeutische Aufnahmeuntersuchung. Sofern nicht alle Untersuchungen an einem Tag stattfinden können, werden diese dann in den darauffolgenden Therapietagen oder nach einer mehrtägigen Pause integriert.

Der Therapieerfolg sollte im Verlauf regelmäßig evaluiert werden. Während sich zur Erfassung der PEM aufgrund ihrer starken Variabilität eine engmaschige Verlaufskontrolle bspw. mittels Symptomtagebuch anbietet, kann eine Reevaluation von PoTS mittels Stehtest in größeren Abständen erfolgen. Ein kombiniertes Aktivitäts- und Symptomtagebuch kann das Selbstmanagement durch Pacing erleichtern. Zur psychologischen Verlaufsevaluierung eignen sich bspw. das Führen eines Belastungstagesbuchs und zur Verlaufsbeurteilung depressiver Symptomatik etablierte Fragebögen wie z. B. der ADS‑L.

Zur wissenschaftlichen Begleitung des Therapiekonzepts erheben wir nach schriftlicher Einverständniserklärung der Patient:innen verschiedene Verlaufsparameter vor und nach der IMST. Ein Votum der Ethikkommission der Ludwig-Maximilians-Universität München liegt vor (18–125) und das Projekt wurde im German Clinical Trials Register (DRKS-ID: DRKS00015141) registriert. Das Programm wurde im Februar 2023 gestartet und es befinden sich bislang acht Patient:innen in Behandlung. Eine systematische Auswertung ist noch nicht erfolgt. Eine umfangreiche Analyse ist für Mitte/Ende 2024 geplant.

## Codierung

Zur Durchführung einer IMST müssen die Kriterien von OPS 8‑91c bzw. 8918 „Interdisziplinäre multimodale Schmerztherapie“ erfüllt sein. Für die Codierung gemäß ICD-10 hat die WHO neben dem Code F45.41 für chronische Schmerzen den Zusatzcode U09.9! für das PCS und G93.3 für ME/CFS festgelegt [28]. In der deutschen ICD-10-GM gibt es noch keine Subcodes für G93.3, wie sie die ICD-10-CM in den USA vorschlägt. Im Falle von Langzeitfolgen einer COVID-19-Impfung (Post-vac-Syndrom), die den postviralen Syndromen und ME/CFS ähneln, ist der ICD-10-Code U12.9 zu wählen. In der Zeit von 4 bis 12 Wochen nach der SARS-CoV-2-Infektion sollte für anhaltende Symptome, die Anlass für eine ärztliche Untersuchung geben, der Code U08.9 eingesetzt werden. Angemessene, spezifische Abrechnungsziffern, die den individuellen therapeutischen Mehraufwand widerspiegeln, gibt es bislang nicht.

## Therapievoraussetzung und Einschlusskriterien

- Leitsymptom chronischer Schmerz und Erfüllung der Kriterien von OPS 8‑91c bzw. 8.918
- Die Begleitsymptomatik inklusive Belastbarkeit sollte eine tagesklinische Behandlung zulassen.
- Wohnortnähe, sodass ein tageweises Pendeln möglich ist

## Therapiekonzept und Behandlungsplan

- 1 Behandlungstag pro Woche mit geplanten 25 bis 30 Behandlungstagen
- 3 bis 4 Module pro Tag (Therapiedauer nach Belastbarkeit), beginnend primär im Einzelsetting
- Ausreichende, mindestens 30-minütige Pause zwischen den Modulen in reizarmer Umgebung, sichergestellt durch Einzelzimmer mit Ruheliege/Bett, gedämpftem Licht, stabiler Temperatur sowie Abwesenheit von starken Gerüchen
- Beschränkung weiterer Stimuli auf ein Minimum durch zeitliche Streckung von Untersuchungen und Instruktion aller Behandelnden hinsichtlich ruhigen Auftretens
- Bei der Wahl der Module werden initial immer ein ärztliches und psychologisches Einzelgespräch sowie ein Entspannungs- bzw. Körperwahrnehmungsverfahren eingeplant.
- Entsprechend der Belastbarkeit folgen im Verlauf weitere Module: u. a. Physio- und Ergotherapie mit Entspannungsverfahren, Atemübungen, Körperwahrnehmungs- und Achtsamkeitstraining, Feldenkrais sowie Liegend-Yoga und Qigong; ebenfalls primär im Einzelsetting.
- Im Verlauf ist dann die Einbettung einzelner Therapiemodule im Gruppensetting zu erwägen.

## Therapiegrundsätze

- Bei allen Maßnahmen muss die individuelle Belastbarkeit beachtet und PEM vermieden werden.
- Implementierung psychoedukativer Maßnahmen und Strategien zum Selbstmanagement, insbesondere zur Vermittlung von angepasstem Energie- und Aktivitätsmanagement (Pacing) als zentrale Form der effektiven Krankheitsbewältigung sowie zur Entwicklung von Coping-Strategien

## Fazit für die Praxis

- Hohe Krankheitslast, hoher Bedarf an therapeutischen Angeboten
- Parallele Pilotierung im laufenden Betrieb einer Schmerztagesklinik gut möglich, auch wenn normalerweise mehrheitlich Gruppenmodule üblich sind
- Das interdisziplinäre Assessment ist zwar aufwendiger (u. a. zusätzliche Fragebögen), dafür besteht aber im therapeutischen Setting ein teils geringerer organisatorischer Aufwand als bei Gruppenplanungen.
- Ein Behandlungstag pro Woche bietet einen guten Kompromiss aus engmaschigem/intensivem Kontakt bei bestmöglicher Vermeidung von PEM und Crashs.
- Diese Behandlungsfrequenz ist auch gut geeignet für die Medikamenteneinstellung sowie schrittweise Anpassung des Aktivierungsniveaus.
- Bislang gibt es keine dem Individualaufwand entsprechende Vergütung.
